# Inversion channel diamond metal-oxide-semiconductor field-effect transistor with normally off characteristics

**DOI:** 10.1038/srep31585

**Published:** 2016-08-22

**Authors:** Tsubasa Matsumoto, Hiromitsu Kato, Kazuhiro Oyama, Toshiharu Makino, Masahiko Ogura, Daisuke Takeuchi, Takao Inokuma, Norio Tokuda, Satoshi Yamasaki

**Affiliations:** 1 Graduate School of Natural Science and Technology, Kanazawa University, Kanazawa, Ishikawa 920-1192, Japan; 2 National Institute of Advanced Industrial Science and Technology, Tsukuba, Ibaraki 305-8568, Japan; 3 Research Laboratories, DENSO CORPORATION, Nisshin, Aichi 470-0111, Japan

## Abstract

We fabricated inversion channel diamond metal-oxide-semiconductor field-effect transistors (MOSFETs) with normally off characteristics. At present, Si MOSFETs and insulated gate bipolar transistors (IGBTs) with inversion channels are widely used because of their high controllability of electric power and high tolerance. Although a diamond semiconductor is considered to be a material with a strong potential for application in next-generation power devices, diamond MOSFETs with an inversion channel have not yet been reported. We precisely controlled the MOS interface for diamond by wet annealing and fabricated p-channel and planar-type MOSFETs with phosphorus-doped n-type body on diamond (111) substrate. The gate oxide of Al_2_O_3_ was deposited onto the n-type diamond body by atomic layer deposition at 300 °C. The drain current was controlled by the negative gate voltage, indicating that an inversion channel with a p-type character was formed at a high-quality n-type diamond body/Al_2_O_3_ interface. The maximum drain current density and the field-effect mobility of a diamond MOSFET with a gate electrode length of 5 μm were 1.6 mA/mm and 8.0 cm^2^/Vs, respectively, at room temperature.

Power devices fabricated using wide-bandgap semiconductors such as SiC and GaN demonstrate better performance than those fabricated using the conventional semiconductor Si and normally off SiC or GaN metal-oxide-semiconductor field-effect transistors (MOSFETs) with an inversion channel have advanced power device technology^1^,^2^,^3^,^4^,^5^,^6^,^7^,^8^. Such power device technology enables an effective utilization of electric power for Shinkansens (bullet trains), airplanes, industrial equipment, medical equipment and so on. Diamond semiconductor has a strong potential for use in the field of high-power electronics because its breakdown electric field and thermal conductivity are higher than those of Si, SiC and GaN. Consequently, diamond-based transistors such as metal-semiconductor field-effect transistors (MESFETs), junction field-effect transistors (JFETs), hydrogen-terminated diamond MOSFETs (H-diamond FETs) and pnp bipolar junction transistors (pnp BJTs) have been developed^9^,^10^,^11^,^12^,^13^,^14^,^15^,^16^,^17^,^18^,^19^,^20^. However, diamond MOSFETs and insulated gate bipolar transistors (IGBTs) with inversion channels have not yet been developed. MOS gates with inversion channels enable a high control of electrical power due to their gate voltage control and the desired threshold voltage can be obtained by controlling the impurity concentration in the bodies. Achieving the desired threshold voltage for devices with an accumulation channel or devices that use the bulk as a channel, such as high-electron-mobility transistors (HEMTs), MESFETs, JFETs and H-diamond FETs, is difficult. Therefore, the normally off characteristics of MOSFETs with an inversion channel are more advantageous than the characteristics of devices with an accumulation channel or devices that use the bulk as a channel when such devices have conduction carrier supplied by the same semiconductor type at their control gate. In addition, in the case of wide-bandgap semiconductor devices, reducing channel resistance is important because the on-resistance of the devices is largely limited by the channel resistance due to the suppressed drift layer resistance. MOSFETs can considerably reduce the channel resistance per unit area when fabricated with a trench gate structure to widen the channel width (*W*_ch_), although the *W*_ch_ of two dimensional channel devices such as HEMTs cannot be widened without increasing the surface area. Moreover, the carrier density of an inversion channel is higher than that of the bulk because wide-bandgap semiconductors have large ionization energies of acceptor and donor impurities.

Because of the aforementioned advantages of MOSFETs, those fabricated with an inversion channel are expected to draw out the maximum performance of semiconducting diamond and represent a substantial advancement in the field of diamond power devices. Therefore, the realization of diamond MOSFETs with an inversion channel is a long-standing research topic. Although diamond MOS capacitors with boron-doped p-type diamond bodies have been reported^21^,^22^,^23^,^24^,^25^, the inversion channel diamond MOSFETs have not yet been reported. Here, we fabricated diamond MOSFETs with a phosphorus-doped n-type diamond body by wet annealing for controlling the MOS interface. In this study, we adopted an n-type diamond body. Here, n-type diamond was selected as a body because its upward bending ability will be advantageous in the inversion mode of FET operation and the band offset of Al_2_O_3_/O-terminated diamond (111) is higher for holes (1.34 eV) than electrons (0.56 eV)^24^,^26^. We operated diamond p-channel MOSFETs with an inversion channel; these diamond MOSFETs exhibit normally off characteristics, clear saturation characteristics and high on/off ratios. We expect the results of this study to represent a major breakthrough in diamond power device technology.

## Results

**Figure 1:**
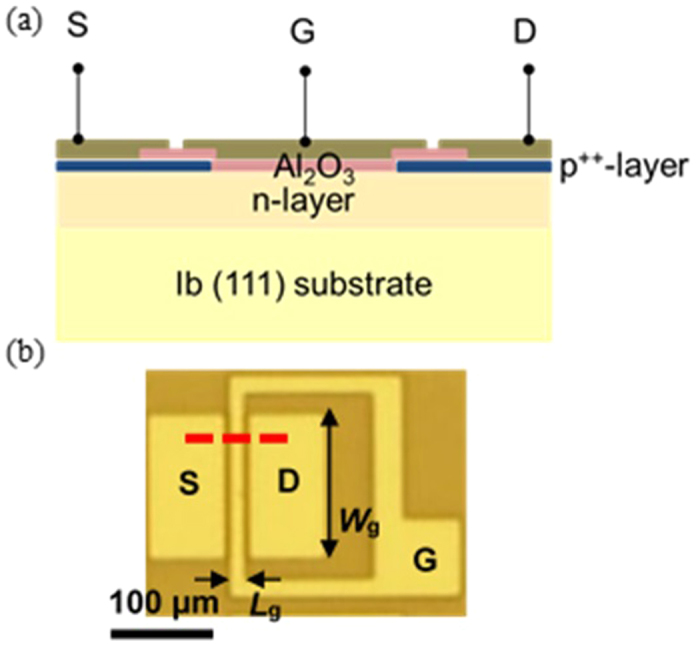
(**a**) Schematic cross-sectional structure and (**b**) top-view optical image of Al_2_O_3_/diamond MOSFET with n-type body. Schematic structure in (**a**) is cross-sectional view along red broken line in (**b**). S, D and G are source, drain and gate contacts, respectively.

In this study, we fabricated diamond MOSFETs using phosphorus-doped n-type diamond as the body, as shown in Fig. 1. Before the deposition of the Al_2_O_3_ layer, we terminated the surface of the n-type diamond body with OH by wet annealing to fabricate a high-quality Al_2_O_3_/O-terminated diamond interface^22^.

**Figure 2:**
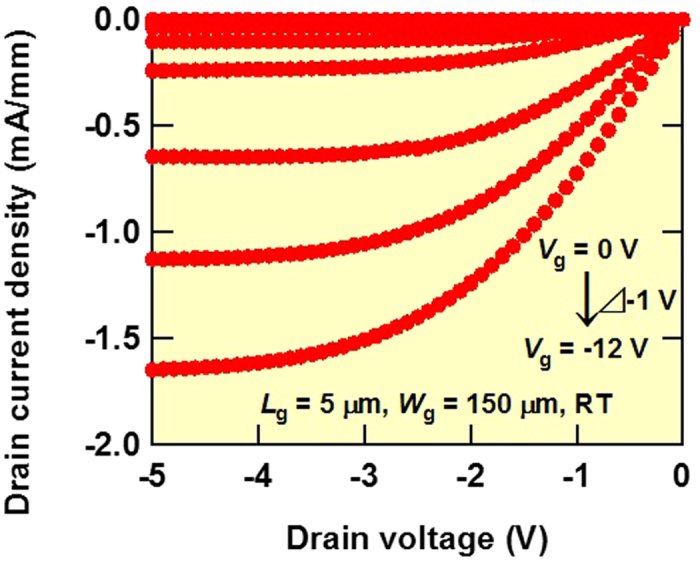
*I*_d_–*V*_ds_ characteristics of diamond MOSFET with *L*_g_ = 5 μm and *W*_g_ = 150 μm at room temperature. Applied *V*_g_ and *V*_ds_ range from 0 to −12 V with a voltage step of −1 V and from 0 to −5 V with a voltage step of −0.1 V, respectively.

Figure 2 shows the drain current (*I*_d_) and drain voltage (*V*_ds_) characteristics at gate voltages (*V*_g_) ranging from 0 to −12 V with a voltage step of −1 V, gate length (*L*_g_) of 5 μm and gate width (*W*_g_) of 150 μm for a diamond MOSFET at room temperature. The MOSFET shows normally off and clear saturation characteristics. *I*_d_ can be well modulated by controlling *V*_g_. Maximum *I*_d_ and drain conductance were −247 μA (drain current density: −1.6 mA/mm) and 110 μS (0.73 mS/mm), respectively. Off *I*_d_ was less than 10^−14^ A at *V*_g_ = −2 V. Therefore, *I*_d_ on/off ratios greater than 10 orders of magnitude were obtained at room temperature. By controlling *V*_g_, 33 of 42 MOSFETs modulated *I*_d_. We also succeeded in controlling *V*_g_ in diamond p-channel MOSFETs using another diamond substrate.

**Figure 3:**
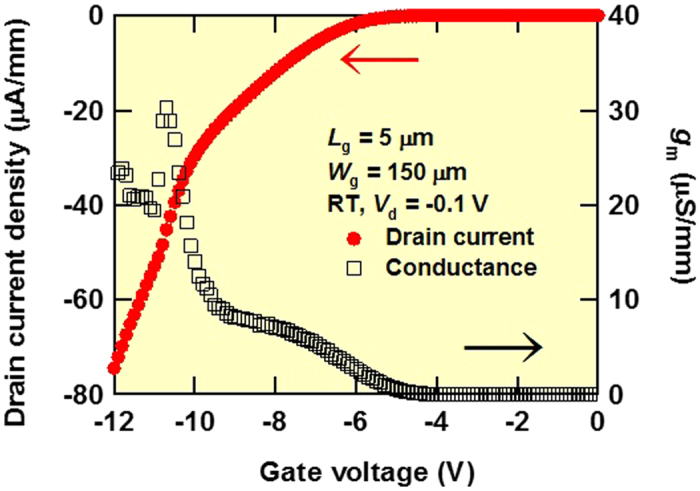
*I*_d_ and *g*_m_ in linear scale vs *V*_g_ of diamond MOSFET with *L*_g_ = 5 μm and *W*_g_ = 150 μm at room temperature. Applied *V*_g_ ranges from 0 to −12 V with a voltage step of −1 V and *V*_ds_ is a constant value of −0.1 V.

**Figure 4:**
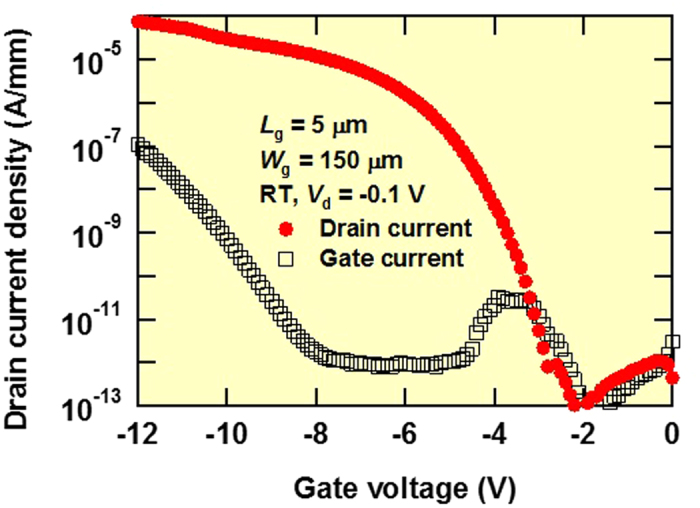
*I*_d_ and *I*_g_ in logarithmic scale vs *V*_g_ of diamond MOSFET with *L*_g_ = 5 μm and *W*_g_ = 150 μm at room temperature. Applied *V*_g_ ranges from 0 to −12 V with a voltage step of −1 V and applied *V*_d_ is a constant value of −0.1 V.

We determined transfer characteristics in the linear region of the *I*_d_–*V*_g_ curve for this MOSFET to obtain the field-effect mobility (*μ*_FE_), subthreshold swing (*SS*) and threshold voltage (*V*_T_). Figure 3 shows *I*_d_ in the linear scale and transfer conductance *g*_m_ vs *V*_g_ characteristics at a low drain voltage (*V*_ds_ = −0.1 V) and *V*_g_ from 0 to −12 V with a voltage step of −0.1 V for a diamond MOSFET with *L*_g_ = 5 μm and *W*_g_ = 150 μm at room temperature. The maximum *g*_m_ was 4.5 μS (30 μS/mm) at *V*_g_ = −10.7 V. *V*_T_ was 6.3 V, as determined from the fitting of the *I*_d_–*V*_g_ curve in the *V*_g_ range from −7 to −9 V. *μ*_FE_ was estimated using the following equation:


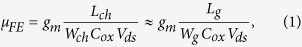


where *L*_ch_ is the channel length and *C*_ox_ is the gate oxide capacitance (*ε* of Al_2_O_3_: 7.3)^22^. Maximum *μ*_FE_ was 8.0 cm^2^/Vs. Figure 4 shows *I*_d_ and the gate current (*I*_g_) in the logarithmic scale vs *V*_g_ characteristics at *V*_ds_ = −0.1 V and *V*_g_ from 0 to −12 V with a voltage step of −0.1 V for a diamond MOSFET with *L*_g_ = 5 μm and *W*_g_ = 150 μm at room temperature. Gate leakage current values were 27 pA/mm at *V*_g_ = −9 V and 110 nA/mm at *V*_g_ = −12 V. *SS* is estimated using the following equation:


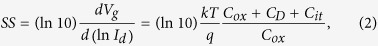


where *k* is the Boltzmann constant, *q* is the electronic charge, *C*_D_ («*C*_ox_) is the depletion layer capacitance, *D*_it_ is the interface-state density and *C*_it_ (=*qD*_it_) is the associated capacitance^27^. The values of *SS* and *D*_it_ were deduced to be 380 mV/dec (from the fitting of the *I*_d_–*V*_g_ curve in the *V*_g_ range from −3.0 to −3.5 V) and approximately 6 × 10^12^ cm^−2^ eV^−1^, respectively.

## Discussion

In general, the inversion channel is checked using the *C*-*V* measurements of the MOS capacitor configuration. For wide-bandgap semiconductors even in SiC^28^, it is difficult to directly measure the inversion capacitance because the opposite carriers are barely excited beyond the bandgap energy. Therefore, we have demonstrated the creation of the inversion channel layer via FET operations with normally off characteristics. When the gate bias was negative, the valence band minimum of the n-type diamond near the gate insulator bend upwards across to the bulk Fermi energy. Holes in the p^+^-type source area can move into the n-type body as minority carriers and towards opposite p^+^-type drain areas, indicating the p-type inversion channel. This observation of Id with normally off characteristics is the direct evidence of a p-type inversion channel layer.

**Figure 5:**
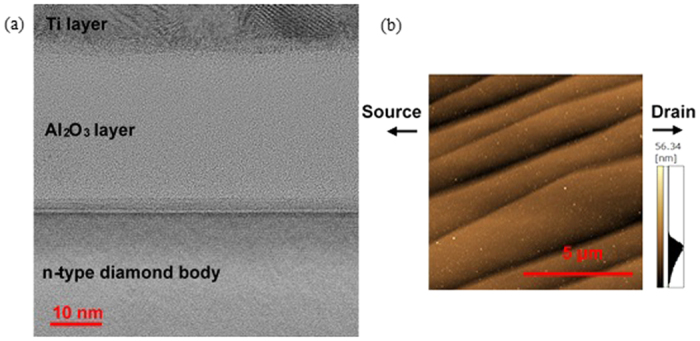
(**a**) TEM image of Al_2_O_3_/diamond interface. (**b**) AFM image of surface of n-type diamond body.

The MOSFETs exhibit a high drain current density compared with previously reported diamond JFETs (0.48 mA/mm) and MESFETs (0.06 mA/mm). This is because of the high bulk resistances of JFETs and MESFETs resulting from the large ionization energies of acceptor (*E*_A_: 370 meV) and donor (*E*_D_: 570 meV) impurities for diamond^10^,^11^. To obtain a high drain current density, i.e., a low on-resistance, the improvement of *μ*_FE_ is necessary. *μ*_FE_ of the present diamond MOSFETs with an inversion channel was 8.0 cm^2^/Vs. Electron *μ*_e_ and hole mobility *μ*_h_ of diamond bulk are greater than 3,000 cm^2^/Vs at room temperature (*μ*_e_ = 7,300 and *μ*_h_ = 5,300 cm^2^/Vs by time-resolved cyclotron resonance and *μ*_e_ = 4,500 and *μ*_h_ = 3,800 cm^2^/Vs by time-of-flight)^29^,^30^. Generally, when a high-quality MOS interface is used, *μ*_FE_ of approximately one half of *μ*_e_ and *μ*_h_ can be obtained in the case of Si MOSFETs. Therefore, *μ*_FE_ greater than 1,000 cm^2^/Vs is expected in diamond MOSFETs. Present *μ*_FE_ is lower than this ideal value because *D*_it_ was very high (~6 × 10^12^ cm^−2^ eV^−1^) for the present Al_2_O_3_/n-type diamond body. Figure 5(a,b) show a transmission electron microscopy (TEM) image of the MOS structure under the gate voltage and an atomic force microscopy (AFM) image of the phosphorus-doped n-type diamond body surface. As shown in Fig. 5(a), although the Al_2_O_3_ layer and n-type diamond body interface appears smooth, the interface exhibited a dark line because the n-type diamond body had some bunching steps across the channel region similar to those shown in Fig. 5(b). Bunching steps cause high *D*_it_ because these steps are not (111) surfaces and are not perfectly OH terminated. Therefore, this partial non-OH termination surface occurs low *μ*_FE_. Atomically flat surface that we previously succeeded is important for reducing *D*_it_ and improving *μ*_FE_^31^. In addition, the quality improvement of the phosphorus-doped n-type diamond body is important for obtaining high *μ*_FE_. The fabrication of high-quality phosphorus-doped n-type diamond bodies is a critical issue in the diamond semiconductor field.

In this study, we could not measure the breakdown voltage of the diamond MOSFETs because *V*_ds_ concentrated at Al_2_O_3_. Introducing a lightly doped layer as an active layer between Al_2_O_3_ and the drain region should result in a high breakdown voltage. This issue is a topic for further investigation.

The present diamond MOSFETs provide a possible path in the realization of ultimate high-power devices.

## Methods

### Sample preparation

Figure 1(a,b) show a schematic cross-sectional structure and top-view optical microscopy image of the planar diamond MOSFET. First, an n-type body was deposited onto a high-pressure, high-temperature (HPHT) synthetic Ib (111) semi-insulating single-crystal diamond substrate by microwave plasma-assisted chemical vapor deposition (CVD). During the growth of the n-type body, the methane concentration, plasma power and chamber pressure were 0.4%, 3.6 kW and 150 Torr, respectively. The thickness and phosphorus concentration of the deposited n-type body were ~10 μm and ~1 × 10^17 ^cm^−3^, respectively. Second, a selective p^+^-type layer was grown on the n-type body through a metal mask (Ti/Au: 10 nm/200 nm) by microwave plasma-assisted CVD. During the growth of the p^+^-layer, the methane concentration, plasma power and chamber pressure were 0.2%, 1200 W and 50 Torr, respectively. The thickness and boron concentration of the selective deposited p^+^-type layer were ~50 nm and ~1 × 10^20 ^cm^−3^, respectively. Third, the sample was annealed in a quartz tube in an electric furnace at 500 °C for 60 min to obtain stable OH surface terminations^22^. The wet annealing was performed under an atmosphere of N_2_ gas bubbled through ultrapure water. The flow of the N_2_ gas was 400 sccm. An Al_2_O_3_ layer was then deposited onto the sample by atomic layer deposition (ALD) at 300 °C. The thickness of the Al_2_O_3_ layer was 34 nm. After the deposition of the Al_2_O_3_ layer, the termination of the diamond surface changed from OH to O, same as that in the ALD mechanism. The gate, drain and source electrodes (Ti/Pt/Au: 30 nm/30 nm/100 nm) were fabricated by photolithography and lift-off, as shown in Fig. 1(b). *L*_g_ and *W*_g_ were 5 μm and 150 μm, respectively. As determined from transfer length model patterns on the same substrate, the contact resistance of the Ti/p^+^-type diamond interface was 2.9 × 10^−6 ^Ωcm^2^ and the leakage current level was less than the detection limit (<10^−14 ^A at ±5 V) for the lateral n-type body and Al_2_O_3_ layer.

### Characterization

The current–voltage (*I*–*V*) characteristics of the MOSFETs were measured using a parameter analyzer (KEITHLEY 4200-SCS). The *I*–*V* measurements were conducted at room temperature in air. AFM measurements were performed using a scanning probe microscope (SHIMADZU SPM-9700). The measurements were conducted in the contact mode over a scanning area of 10 × 10 μm^2^ using a Si cantilever (Hitachi High-Tech Science Corp. SI-DF20). Cross-sectional TEM images were obtained using TEM system of JEOL JEM-ARM200F operated at an acceleration voltage of 14.5 keV.

## Additional Information

**How to cite this article**: Matsumoto, T. *et al*. Inversion channel diamond metal-oxide-semiconductor field-effect transistor with normally off characteristics. *Sci. Rep.* **6**, 31585; doi: 10.1038/srep31585 (2016).
